# Reliability of the evidence to guide decision-making in foot ulcer prevention in diabetes: an overview of systematic reviews

**DOI:** 10.1186/s12874-022-01738-y

**Published:** 2022-10-20

**Authors:** Fay Crawford, Donald J. Nicolson, Aparna E. Amanna, Marie Smith

**Affiliations:** 1 The School of Medicine, The University of St Andrews, North Haugh, St Andrews, KY16 9TF UK; 2 NHS Fife, Kirkcaldy, UK

**Keywords:** Overview, Systematic reviews, Evidence-based health care

## Abstract

**Background:**

Reliable evidence on the effectiveness of interventions to prevent diabetes-related foot ulceration is essential to inform clinical practice. Well-conducted systematic reviews that synthesise evidence from all relevant trials offer the most robust evidence for decision-making. We conducted an overview to assess the comprehensiveness and utility of the available secondary evidence as a reliable source of robust estimates of effect with the aim of informing a cost-effective care pathway using an economic model. Here we report the details of the overview. [PROSPERO Database (CRD42016052324)].

**Methods:**

Medline (Ovid), Embase (Ovid), Epistomonikos, Cochrane Database of Systematic Reviews (CDSR), Database of Abstracts of Reviews of Effectiveness (DARE), and the Health Technology Assessment Journals Library were searched to 17th May 2021, without restrictions, for systematic reviews of randomised controlled trials (RCTs) of preventive interventions in people with diabetes. The primary outcomes of interest were new primary or recurrent foot ulcers. Two reviewers independently extracted data and assessed the risk of bias in the included reviews.

**Findings:**

The overview identified 30 systematic reviews of patient education, footwear and off-loading, complex and other interventions. Many are poorly reported and have fundamental methodological shortcomings associated with increased risk of bias. Most concerns relate to vague inclusion criteria (60%), weak search or selection strategies (70%) and quality appraisal methods (53%) and inexpert conduct and interpretation of quantitative and narrative evidence syntheses (57%). The 30 reviews have collectively assessed 26 largely poor-quality RCTs with substantial overlap.

**Interpretation:**

The majority of these systematic reviews of the effectiveness of interventions to prevent diabetic foot ulceration are at high risk of bias and fail to provide reliable evidence for decision-making. Adherence to the core principles of conducting and reporting systematic reviews is needed to improve the reliability of the evidence generated to inform clinical practice.

**Supplementary Information:**

The online version contains supplementary material available at 10.1186/s12874-022-01738-y.

## Background

Diabetes mellitus is a major global public health problem. In 2019, 463 million adults around the world were living with diabetes and projections predict an increase in prevalence to 578 million by 2030 and 700 million by 2045 [1]. In the UK alone it is estimated that 5 million people will have diabetes by 2030 [2]. People with diabetes are more at risk of developing foot problems with those affected experiencing higher rates of foot ulceration, lower-limb amputation and premature death [3, 4]. The healthcare costs of diabetic foot ulcers and amputations to the NHS in England has been estimated at between £837 and £962 million, almost 1% of the NHS budget, with more than 90% of that expenditure related to ulceration [3].

Reliable evidence on the clinical effectiveness of preventive interventions is imperative to design effective care pathways that can reduce the risk of foot ulceration and its adverse consequences for people with diabetes and the associated healthcare costs. As part of a wider research project to develop an evidence-based care pathway we sought to obtain numerical estimates of effect from randomised controlled trials (RCTs) of interventions to prevent diabetic foot ulceration as RCTs have the advantage over other study designs when evaluating interventions because only a randomly allocated control group comparison can prevent systematic differences at baseline influencing the results and support reliable claims about cause and effect [5, 6].

Systematic review methods are widely used to summarise the evidence generated by multiple individual primary studies of alternative interventions to support decision-making and inform clinical practice, guidelines and health policy [6, 7]. Well-conducted systematic reviews based on explicit methods that identify, appraise and summarise the findings from all relevant primary studies of the same and alternative interventions can determine which results are sufficiently reliable to inform practice and provide more accurate estimates of effect than individual studies alone. It is however becoming increasingly common to find multiple systematic reviews in the published literature that address the same clinical questions [8, 9]. In this situation an overview can provide a comprehensive summary of the evidence base and reduce the research duplication and waste that is generated by conducting unnecessary additional reviews [10, 11]. Overviews have a similar structure and methodology to systematic reviews but include reviews rather than primary studies [12].

Several published systematic reviews of preventative interventions for foot ulceration in diabetes are known to exist, some of which reach conflicting conclusions [13, 14]. We conducted an overview to assess the comprehensiveness and utility of the available secondary evidence as a reliable source of robust estimates of effect with the aim of informing a cost-effective care pathway using an economic model, based on numerical data [5]. Although we identified 19 systematic reviews (one of which had been updated) limitations in scope, overlap and quality meant we had to undertake an additional systematic review in order to make the best possible use of the available data [14]. The purpose of this overview is to update the original searches for eligible reports and to consider the quality and reliability of systematic reviews of preventative interventions for foot ulceration in diabetes.

The overview protocol was registered on the PROSPERO Database (registration number: CRD42016052324).

## Methods

The literature search, selection and appraisal methods are summarised here and reported in detail elsewhere [5].

### Search strategy

A librarian (MS) developed strategies to identify systematic reviews in Medline OVID and Embase OVID (initially from inception to December 2019 then re-run to update the searches until 17th May 2021) without restrictions. The first searches were de duplicated using RefWorks. The electronic search strategies were informed by the strategies reported elsewhere [11] and include methodological search terms (see Additional file 1: supplementary files). The Cochrane Database of Systematic Reviews (CDSR), the Database of Abstracts of Reviews of Effectiveness (DARE), and the Health Technology Assessment (HTA) Journals Library and (for the update search only, Epistomonikos) were also searched. Systematic reviews in progress were identified via PROSPERO (https://www.crd.york.ac.uk/prospero/) and checked for subsequent completion or publication. Reference lists in all eligible reviews were browsed for additional relevant reviews. Additional data and clarifications about their reviews were sought from review authors.

### Eligibility criteria

Systematic reviews of RCTs of interventions to prevent foot ulceration in people with type 1 or type 2 diabetes whether at high, medium, or low risk, with or without a history of foot ulceration but no existing foot ulcers at baseline were eligible for inclusion. The outcomes of interest were incident primary or recurrent foot ulcers and Lower Extremity Amputations (LEA) derived from RCTs comparing single-component or complex interventions (comprising several interacting components provided together) with standard care or alternative interventions. We excluded reviews of surgical procedures. Systematic reviews that included RCTs and other study designs were eligible for inclusion but only data from the relevant RCTs was used for the purpose of the overview.

### Selection and data extraction

One reviewer (DJN or FC) screened all titles and abstracts to identify potentially relevant reviews with a second reviewer (FC or HMc) screening a 10% random sample to minimise the risk of errors of judgement. Reviewers working in pairs (DJN, AEA, FC or HMc) independently assessed the selected full text articles for eligibility and resolved disagreements in discussion with a third reviewer. Reviewers (DJN, AEA, FC or HMc) independently extracted data from the included reviews using a bespoke data extraction tool and resolved disagreements through discussion.

### Quality assessment

Reviewers working in pairs (DJN, AEA, FC or HMc) independently assessed the risk of bias in the included reviews using the Risk of Bias in Systematic Reviews (ROBIS) tool and reached agreement by discussion [12]. Concerns with the process of reviews are assessed using 4 domains; (i) study eligibility criteria, (ii) the identification and selection of studies, (iii) data collection and study appraisal and (iv) synthesis and findings.

## Results

**Fig. 1:**
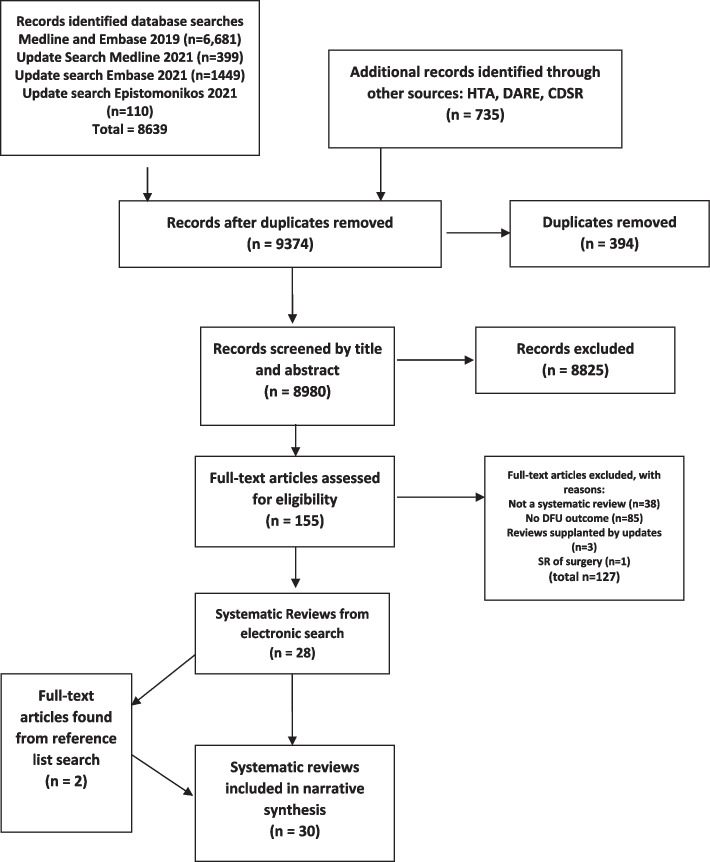
Flow diagram. Additional searches: CDSR – Cochrane Database of Systematic Reviews; DARE – Database of Abstracts of Reviews of Effectiveness; HTA – Health Technology Assessment Journals Library

A diagram showing the flow of information through the process of identifying and selecting reviews for inclusion in the overview is presented in Fig. 1.

### Included reviews

Thirty-two reviews met the criteria for inclusion in the overview [13–44]. Two were updates of previously published reviews and the earlier versions were excluded to avoid the double-counting of data [31, 33]. Of the 30 reviews, 14 included only RCTs [13–26] and 16 included RCTs together with various other study designs [27–30, 32, 34–44]. The reviews were published between 1998 and 2021 in professional or scientific journals, four in the Cochrane Library, one was published in the UK National Institute of Health Research (NIHR) Health Technology Assessment (HTA) journals library and one for the Agency for Healthcare Research and Quality (AHRQ), USA [15, 16, 19, 20, 24, 41]. Other key characteristics of the included reviews are summarised in Table 1.

**Table 1:** Characteristics of included reviews

**Author (date) Funding**	**Objective**	**Eligibility Criteria**					**Evidence relevant to the overview**
		**Population**	**Interventions**	**Comparators**	**Study designs**	**Outcomes**	
**Reviews of patient education interventions**							
Adiewere (2018) [13] UK Funding: The Independent Diabetes Trust (UK)	To examine the effectiveness of patient education in preventing and reducing the incidence or recurrence of adult diabetic foot ulceration and amputation	Type I or type II diabetes mellitus or those with diabetic foot ulcers, aged ≥18 years; ulceration risk not specified	Intervention focused on patient education	Not specified	RCTs	Incidence of diabetic foot ulcers; amputation rates	3 RCTs (Additional file 2: Supplementary references S2–S4) (*n* = 423) Ulcer risk: high Outcomes: ulceration; amputations Meta-analysis
Ahmad Sharoni (2016) [29] Malaysia Funding: Not reported	To assess the effectiveness of health education programmes to improve foot self-care practices and foot problems among older people with diabetes	Older people with diabetes, average age ≥ 60 years; ulceration risk not specified	Educational programmes in relation to diabetes foot selfcare that included teaching, coaching, discussion, demonstration, and assessment, conducted by medical personnel	Not specified	Intervention studies (e.g. RCTs, non-randomised controlled study); studies with or without a control group	Primary outcome: diabetes foot self-care Secondary outcome: foot problems (neuropathy, lesion, ulcer, amputation, foot disability, callus, tinea pedis)	1 RCT (Additional file 2: Supplementary reference S2) (*n* = 172) Ulcer risk: high Outcomes: ulceration; amputations Narrative summary
Dorresteijn (2014) [15] Netherlands Funding: Cochrane review; no additional information	To assess the effects of patient education on the prevention of foot ulcers in patients with diabetes mellitus	Type 1 or type 2 diabetes mellitus, ≥18 years; ulceration risk not specified	Educational programmes or programmes that include education to reduce the incidence of foot ulceration; foot-care education as part of a larger educational programme or a more comprehensive diabetic foot programme	All types of control intervention	RCTs	Primary outcomes: foot ulceration or ulcer recurrence; amputation Secondary outcomes: Callus development; resolution of callus; fungal infection; number and duration of hospital admissions for diabetic foot problems; foot care knowledge scores; patients’ behaviour assessment scores	2 RCTs (Additional file 2: Supplementary references S1, S2) (*n* = 225) Ulcer risk: high Outcomes: ulceration; amputations Narrative summary
He (2013) [17] China Funding: Not reported	To assess the effectiveness of intensive versus routine education on diabetes mellitus for preventing diabetic foot ulcer	Type I or type II diabetes mellitus, ≥18 years, without current diabetic foot ulcer; ulceration risk not specified	Intensive diabetic education (any time, manner and duration of treatment)	Routine diabetes education	RCTs	Primary outcomes: incidence of diabetic foot ulcers; amputation rate Secondary outcomes: diabetes knowledge; quality of life	2 RCTs (Additional file 2: Supplementary references S1, S2) (*n* = 225) Ulcer risk: high Outcomes: ulceration; amputations Meta-analysis
**Reviews of psychosocial interventions**							
**Author (date) Funding**	**Objective**	**Eligibility Criteria**					**Evidence relevant to the overview**
		**Eligibility Criteria**	**Evidence relevant to the overview**	**Author (date) Funding**	**Objective**	**Eligibility Criteria**	
McGloin (2021) [24] Ireland Funding: NIHR (UK) via Cochrane Wounds Group infrastructure funding	To evaluate the effects of psychological interventions on healing and recurrence of diabetic foot ulceration	People with a diabetic foot ulcer or a history of diabetic foot ulcer, ≥18 years; in any care setting	Psychological interventions: CBT; cognitive therapy; psychodynamic therapy; counselling; family systems or systemic therapy; other, as provided by a facilitator.	Standard care, another psychological intervention, education on healing and/or recurrence	RCTs, quasi-RCTs	Primary outcomes: complete wound healing, time to recurrence, number of recurrences Secondary outcomes: amputations (major or distal), health-related quality of life, self-efficacy, cost	1 RCT (*n* = 41) (Additional file 2: Supplementary reference S25) Ulcer risk: High Outcomes: ulceration Analysis; narrative summary
Norman (2020) [23] UK Funding: NIHR (UK)	Summarise the evidence for the effectiveness of psychosocial interventions to promote healing and/or reduce the occurrence of foot ulceration in people with diabetes	*Type 1 or 2 diabetes with or without active foot ulceration; any ulceration risk level*	Any individual or group psychological, behavioural or social intervention alone or in combination (eg CBT, motivational interviewing counselling, psychological therapy, social support, mindfulness)	Any, including alternative interventions, usual care, no treatment	RCTs	Primary outcomes: complete healing, ulceration *Secondary outcomes: wound healing rate, amputation, foot care knowledge, adverse events, mortality, psychological outcomes, health related quality of life*	6 RCTs (*n* = 824) (Additional file 2: Supplementary references S1–S4, S12, S14) Ulcer risk: All levels Outcomes: ulceration Analysis: narrative summary
Binning (2019) [27] UK Funding: Not reported	To determine whether motivational interviewing has been found to be an effective intervention to improve adherence behaviours for the prevention of diabetic foot ulceration	Diabetes of any type, aged ≥18 years, classified as “at risk” of developing diabetic foot ulceration as defined by the IWGDF, with current or recurrent ulceration or a co-existing risk factor	Motivational interviewing or a motivational approach as the sole intervention or as an intervention component; interventions solely aimed at improving knowledge and skills were excluded	All types of control intervention were accepted	Not pre-specified beyond studies without comparator groups were eligible if they were prospective with a before-and-after study design	*A new episode of ulceration and/or at least one behavioural outcome measure*	1 RCT (Additional file 2: Supplementary reference S4) (*n* = 131) Ulcer risk: high Outcomes: ulceration Narrative summary
**Reviews of footwear and off-loading interventions**							
**Author (date) Funding**	**Objective**	**Eligibility Criteria**					**Evidence relevant to the overview**
		**Population**	**Interventions**	**Comparators**	**Study designs**	**Outcomes**	
Ahmed (2020) [38] Australia Funding: None	Evaluate the evidence for footwear and insole features that reduce pathological plantar pressures and diabetic neuropathy ulceration at the plantar forefoot in people with diabetic neuropathy	Diabetes, > 18 years; with or without neuropathy and foot deformity, history of plantar forefoot ulcers but no Charcot foot, history of heel ulcer or active foot ulcers	Footwear or insoles as a long-term offloading intervention; conventional materials and manufacturing techniques; closed-in footwear	Not specified	All study designs except systematic reviews and case reports	Outcomes: (re)-occurrence of forefoot ulcer or change in forefoot plantar pressure	5 RCTs (*n* = 888) (Additional file 2: Supplementary references S7–S10, S19) Ulcer risk: High Outcomes: ulceration Analysis: Narrative synthesis
Bus (2015) [32] Netherlands Funding: Not reported	To assess the effectiveness of footwear and offloading interventions to prevent or heal foot ulcers or reduce mechanical pressure in patients with diabetes	Type I or type II diabetes mellitus; ulceration risk not specified	Casting, footwear, surgical offloading, and other offloading techniques	Not specified	Systematic reviews, meta-analyses, RCTs, non-randomised controlled trials, case–control studies, cohort studies, controlled before-and-after studies, interrupted time series, prospective and retrospective uncontrolled studies, cross-sectional studies, case series	Ulcer prevention, ulcer healing, pressure reduction	7 RCTs (Additional file 2: Supplementary references S5–S11) (*n* = 1554) Ulcer risk: high Outcomes: ulceration Analysis: Narrative summary
Collings (2021) [40] UK Funding: NIHR (UK)	To identify the best footwear and insole design features for offloading the plantar surface of the foot to prevent foot ulceration in people with diabetic peripheral neuropathy	Type 1 or type 2 diabetes mellitus, > 18 years; peripheral neuropathy, nonulcerated feet, no major amputation of the foot or Charcot arthropathy	Therapeutic footwear and/or insole	Therapeutic footwear and/or insole design feature compared with another or no intervention	RCTs, non-randomised controlled trials, quasi-experimental, before and after studies, prospective and retrospective cohort studies, analytical cross-sectional studies	Primary outcome: foot ulcer incidence Other outcomes: kinetic, kinematic or clinical measures of plantar foot loading/offloading, side effects or adverse events	7 RCTs (*n* = 1357) (Additional file 2: Supplementary references S5–S10, S18, S19) Ulcer risk: High Outcomes: ulcer Analysis: Narrative summary of RCT data
Heuch (2016) [28] Australia Funding: Not reported	Review question: what is the effectiveness of methods of offloading in preventing primary DFUs in adults with diabetes?	Diabetes mellitus, ≥18 years, no history of foot ulceration	All offloading methods and strategies to prevent foot ulcers	Alternative offloading methods; all other comparators, including traditional treatment by a podiatrist	All quantitative study designs, including RCTs, quasi-experimental studies, cohort studies, case-control studies, and descriptive studies including case series and before-and-after studies	Primary outcome: prevention of primary diabetic foot ulcers Secondary outcome: indications of changes in pressure on the skin of the foot	0 RCTs
Healy (2013) [34] UK Funding: Not reported	To examine the effectiveness of footwear as an intervention for prevention of diabetic foot ulcers or the reduction of biomechanical risk factors for ulceration	Type I or type II diabetes mellitus, adults; ulceration risk not specified	Footwear as a preventive intervention	Alternative types of footwear	RCTs, quasi-experimental and observational studies with a control group or a repeated measure design	Ulceration/re-ulceration rates or biomechanical risk factors for ulceration (callus and plantar pressure measurement)	2 RCTs (Additional file 2: Supplementary references S5, S6) (*n* = 469) Ulcer risk: high Outcomes: ulceration Narrative summary
Paton (2011) [35] UK Funding: Not reported	To evaluate the effectiveness of insoles used for the prevention of ulcer in the neuropathic diabetic foot	Type I or type II diabetes mellitus, diagnosed with neuropathy, free from ulceration at study entry; ulceration risk not specified	Insoles	Not specified	RCTs, non-randomised control trials, follow-up/longitudinal studies	Primary outcome: time to ulceration Other outcomes: pressure measurement, patient-based response/patient perception, cost	1 RCT (Additional file 2: Supplementary reference S5) (*n* = 69) Ulcer risk: high Outcomes: ulceration Narrative summary
Maciejewski (2004) [36] USA Funding: Department of Veterans Affairs (USA)	To review the evidence for the effectiveness of therapeutic footwear in preventing re-ulceration in people with diabetes and foot risk factors	Individuals with diabetes and foot risk factors	Off the shelf therapeutic shoes with custom or generic inserts or custom shoes with custom inserts; used with other interventions or footwear reimbursement as the primary intervention	Not specified	RCTs, non-randomised controlled trials, analytic studies, descriptive studies	Prevention of re-ulceration	2 RCTs (Additional file 2: Supplementary references S5, S6) (*n* = 469) Ulcer risk: high Outcomes: ulceration Narrative summary
Spencer (2000) [20] UK Funding: Cochrane review; no external sources of support	To assess the effectiveness of pressure relieving interventions in the prevention and treatment of diabetic foot ulcers	Type 1 or Type 2 diabetes mellitus, with (treatment trials) or without (prevention trials) foot ulcers; ulceration risk not specified	Relief or redistribution of pressure in the neuropathic and/or neuroischaemic diabetic foot e.g. callus removal, orthoses including insoles, bespoke and customised shoes and casts	Not specified	RCTs	Time to complete healing or proportion of ulcer(s) completely healed in trial time, healing rates, recurrence rates, new ulceration, complications and morbidity, patient satisfaction, quality of life, presence of callus, cost effectiveness, cost benefit	1 RCT (Additional file 2: Supplementary reference S5) (*n* = 69) Ulcer risk: high Outcomes: ulceration Narrative summary
**Reviews of complex Interventions**							
**Author (date) Funding**	**Objective**	**Eligibility Criteria**					**Evidence relevant to the overview**
		**Population**	**Interventions**	**Comparators**	**Study designs**	**Outcomes**	
Hoogeveen (2015) [16] Netherlands Funding: Cochrane review; NIHR (UK) via Cochrane Wounds Group infrastructure funding	To assess the effectiveness of complex interventions in the prevention of foot ulcers in people with diabetes mellitus	Type 1 or type 2 diabetes mellitus, ≥18 years; ulceration risk not specified	Complex intervention defined as an integrated care approach, combining ≥2 prevention strategies on at least two different levels of care (patient, healthcare provider and/or structure of health care)	Any comparison including single interventions, usual care, and alternative complex interventions	RCTs	Primary outcomes incidence of foot ulceration; partial or total amputation rates Secondary outcomes: callus development; resolution of callus; number and duration of hospital admissions for diabetes related foot problems; foot care knowledge scores; patients’ behaviour assessment scores; adverse events; costs	3 RCTs (Additional file 2: Supplementary references S12–S14) *n* = 2455) Ulcer risk: All levels Outcomes: ulceration; amputations Narrative summary
Blanchette (2020) [39] Canada Funding: None	What is the effect of contact with a podiatrist and their interventions in an MDT context on LEA and DFU in people with diabetes?	Type 1 or type 2 diabetes mellitus, ≥18 years; ulceration risk not specified	Educational prevention, *foot care, offloading, infection control, wound care and surgical strategies delivered by a podiatrist in a multidisciplinary context or MDT programme*	Interventions or treatments without an MDT context	RCTs, prospective or retrospective cohorts, comparative cohorts before and after	Primary outcomes: DFU or LEA occurrence Secondary outcomes: mortality/survival, DFU or LEA reoccurrence, other complications (infection), healthcare utilization, patient satisfaction	0 RCTs
Mayfield (2000) [37] USA Funding: Not reported	To evaluate the evidence supporting the Semmes-Weinstein monofilament and other threshold testing in preventing ulcers and amputation in people with diabetes	People with diabetes; ulceration risk not specified	Semmes-Weinstein monofilament or another threshold neuropathy assessment method that could be conducted in a primary care setting	A reference standard of foot ulceration or amputation (for diagnostic evaluations)	Not specified	Ulceration, amputation	1 RCT (Additional file 2: Supplementary reference S13) (*n* = 1997) Ulcer risk: all levels Outcomes: ulceration; amputations Narrative summary
**Reviews of telehealth and temperature monitoring interventions**							
**Author (date) Funding**	**Objective**	**Eligibility Criteria**					**Author (date) Funding**
		**Population**	**Interventions**	**Comparison**	**Study Designs**	**Outcomes**	
da Silva (2020) [43] Brazil Funding: Not Reported	Assess the effect of mobile technologies as a tool in diabetic foot prevention and diagnosis in people with diabetes mellitus	Type 1 or type 2 diabetes mellitus; ulceration risk not specified	Mobile technology aimed at diabetic foot prevention, foot self-care, or diagnosis	NS	NS	NS	0 RCTs
Hazenberg (2020) [42] The Netherlands Funding: Not Reported	To assess the psychometric properties, feasibility, effectiveness, costs, and current limitations of telehealth and telemedicine approaches for prevention and management of diabetic foot disease	Diabetes mellitus with or at risk of developing a foot ulcer (risk defined according to the IWGDF as having peripheral neuropathy, with or without foot deformities, peripheral artery disease or lower-extremity amputation and/or a history of foot ulceration)	Any telehealth or telemedicine application, or medical tool that may potentially serve as a telehealth or telemedicine application	NS	RCTs, non-randomised trials, case-control studies, cohort studies, cross-sectional studies, case series, case reports, qualitative research	Validity, reliability, feasibility, effectiveness, costs in the outcome categories of monitoring, prevention, or treatment of diabetic foot disease	4 RCTs (*n* = 524) (Additional file 2: Supplementary references S21–S23, S25) Ulcer risk: High Outcomes: ulceration Analysis: Narrative summary
Ena (2021) [26] Spain Funding: None	To determine the effectiveness of the daily measurement of foot temperature in 6 points to prevent the occurrence of foot ulcers in patients with diabetes	Type 2 diabetes, at high risk of developing foot ulcers (history of neuropathy, deformity of the feet, or previous ulcer)	Twice daily monitoring of the temperature of the sole of the foot in 6 different locations along with the standard of care (use of insoles or orthopaedic footwear, education on diabetic foot prevention, routine foot care)	Standard of foot care (education, self-care practices, periodic clinical visits)	RCTs	Primary outcome: incidence of new foot ulcers (proportion of patients who developed a foot ulcer during follow-up)	4 RCTs (*n* = 524) (Additional file 2: Supplementary references S21–S23, S25) Ulcer risk: High Outcomes: ulceration Analysis: meta analysis
**Reviews of physical activity and exercise interventions**							
**Author (date) Funding**	**Objective**	**Eligibility Criteria**					**Author (date) Funding**
		**Population**	**Intervention**	**Comparison**	**Study design**	**Outcome**	
Matos (2018) [44] Portugal Funding: NORTE2020, European Regional Development Fund	To analyse the effects of exercise and physical activity interventions on diabetic foot outcomes	A diagnosis of diabetes or diabetic peripheral neuropathy, polyneuropathy or DFU	Any form of supervised physical activity at home or a care centre	Daily life physical activity and/or usual foot care education	Controlled clinical trials	*Diabetic foot outcomes*	1 RCT (*n* = 79) (Additional file 2: Supplementary reference S24) Ulcer risk: all levels Outcomes: ulceration Analysis: narrative summary
**Reviews of assorted preventive interventions**							
**Author (date) Funding**	**Objective**	**Eligibility Criteria**					**Evidence relevant to the overview**
		**Population**	**Interventions**	**Comparators**	**Study designs**	**Outcomes**	
Alahakoon (2020) [25] Australia Funding: James Cook University Strategic Research Investment Fund and other listed sources	Perform a systematic review and meta-analyses of RCTs of home foot temperature monitoring, education and offloading footwear for reducing the incidence of diabetes-related foot ulcers	Diabetes; IWGDF risk categories 2 or 3 without active diabetes-related foot ulcers	Home foot temperature monitoring, patient education, offloading footwear	Control group not receiving the intervention under study	RCTs	Primary outcome: DFU incidence (full thickness wound on the foot) Secondary outcomes: minor, major and total amputations	17 RCTs (Additional file 2: Supplementary references S1–S10, S14, S19–S23, S25) (*n* = 2729) Ulcer risk: High Outcomes: ulceration, amputations Analysis: Meta analysis
Arad (2011) [18] USA Funding: Not reported	To systematically assess RCTs regarding possible methods to prevent diabetic foot ulcers	People with diabetes, at risk of ulceration, with or without a history of previous foot ulcers	Primary and secondary prevention methods	Not specified	RCTs	Primary outcome: diabetic foot ulcers	9 RCTs (Additional file 2: Supplementary references S2, S5, S6, S9, S12, S13, S21–S23) (*n* = 3816) Interventions: patient education (1 trial); footwear (3 trials); complex (2 trials); other (3 trials) Ulcer risk: high Outcomes: ulceration; amputations Narrative summary
Crawford (2020) [14] UK Funding: Health Technology Assessment	To systematically review data from RCTs of interventions used to prevent foot ulcerations in diabetes.	people of any age with a diagnosis of type 1 or type 2 diabetes, with or without a history of ulceration, but free from foot ulceration at trial entry.	Simple interventions (e.g. education aimed at individuals with diabetes or physicians, or the provision of footwear) and complex interventions.	Standard Care or active treatments	RCTs	Incident or recurrent foot ulcers; also sought data on amputation; mortality; gangrene; infection; adverse events; harms; time to ulceration; quality of life; timing of screening; self-care; hospital admissions; psychological; and adherence to therapy	22 RCTs (Additional file 2: Supplementary references S1–S15, S17, S21–S26) (*n* = 5410) Outcomes: ulceration, amputation Ulcer risk: all levels Analysis: meta analysis
Dy (2017) [41] USA Funding: AHRQ	To assess benefits and harms of interventions for preventing diabetic peripheral neuropathy complications and treatment of symptoms	Type 1 or type 2 diabetes; ≥18 years; at risk for peripheral polyneuropathy	Pharmacologic (glucose lowering) focused on glucose control; Nonpharmacologic (foot care, surgical interventions, dietary strategies, lifestyle interventions, exercise and balance training) and surgical	Active interventions, usual care/placebo	Systematic reviews, RCTs, non-randomised studies with concurrent comparison groups	Incident or recurrent foot ulcer, falls, perceived fall risk, amputation, HRQoL, physical activity level, harms	18 RCTs (*n* = 2778) (Additional file 2: Supplementary references S1, S3—S11, S14, S15, S17, S21–S25) Ulcer risk: all levels Outcomes: ulcer Analysis: meta analysis.
O’Meara (2000) [19] UK Funding: Health Technology Assessment Board (UK)	To estimate the clinical and cost effectiveness of interventions for the prevention and treatment of diabetic foot ulcers	Diabetes mellitus, with a foot ulcer (treatment studies) or at risk of foot ulceration (prevention studies); ulceration risk not specified	Any intervention for the prevention and/or treatment of diabetic foot ulcers	Not specified	RCTs, non-RCTs with a contemporaneous control	Development or resolution of callus; incidence of ulceration; ulcer healing; ulcer recurrence rates; side effects; amputation rates	4 RCTs (Additional file 2: Supplementary references S5, S12, S13, S26) (*n* = 2622) Interventions: footwear (1 trial); complex (2 trial); other (1 trial) Ulcer risk: all levels Outcomes: ulceration; amputations Narrative summary
Mason (1999) [21] UK Funding: NHS Executive and British Diabetic Association	To evaluate the role of preventative strategies in reducing foot ulcers in patients with Type 2 diabetes mellitus, both in the general population and those identified to be at a raised risk	Type I or type II diabetes mellitus; ulceration risk not specified	Studies that addressed some aspect of screening, management, prevention or education relating to the foot care of people with diabetes	Not specified	RCTs, studies of lesser design	Not specified	3 RCTs (Additional file 2: Supplementary references S5, S12, S13) (*n* = 2462) Ulcer risk: all levels Outcomes: ulceration; amputations Narrative summary
Kaltenthaler (1998) [22] UK Funding: Not Reported	To review evidence on the effectiveness of interventions (including prevention) for diabetic foot ulcers	Diabetes; ulceration risk not specified	Prevention, multi-disciplinary education and support, treatments including topical applications, dressings, surgery, antibiosis, growth substances, hyperbaric oxygen, drug therapy, wound grafting, footwear and contact casts	Not specified	RCTs	Diabetic foot ulcers	2 RCTs (Additional file 2: Supplementary references S5, S12) (*n* = 464) Ulcer risk: high Outcomes: ulceration Narrative summary
van Netten (2020) [30] update of van Netten (2016) [31] Netherlands Funding: Not reported	To investigate the effectiveness of interventions to prevent first and recurrent foot ulcers in persons with diabetes who are at-risk for ulceration and do not have a current foot ulcer	Type I or type II diabetes mellitus, at risk of foot ulceration defined as presence of peripheral neuropathy, with or without a foot deformity or peripheral artery disease, or a history of foot ulcers or amputation of the foot or leg	Preventive interventions aimed at improvement in care, education of healthcare professionals, patient self- management, and medical interventions	Not specified	Systematic reviews, meta-analyses, RCTs, non-randomised controlled trials, case–control studies, controlled cohort studies, before-and-after studies, interrupted time series, prospective and retrospective non-controlled studies, cross-sectional studies, case series	Primary outcomes: first diabetic foot ulcer, recurrent diabetic foot ulcer	20 RCTs (Additional file 2: Supplementary references S1–S11, S14–S17, S21–S25) (*n* = 2968) Outcomes: ulceration; amputations Narrative summary

Overall, the 30 reviews included a total of 26 RCTs relevant to the overview (See Additional file 2: Supplementary references S1–S26). The majority of the RCTs were included in more than one review, only three being included only once (see Additional file 2: Supplementary references S16, S18, S20).

### Risk of bias

The ROBIS assessment results are summarised in Table 2. Six were judged to have a low risk of bias in all four domains assessed using the ROBIS tool [15, 16, 19, 20, 23, 24]. Nineteen reviews (65%) were judged to be at high risk of bias [13, 17, 18, 21, 22, 27–29, 32, 34–40, 42–44]. The most common reasons for concern about bias in the reviews related to the lack of clarity in eligibility criteria specification (60%) [13, 17, 18, 21, 22, 27–29, 35–44] methods used to identify and select eligible studies (70%) [13, 14, 17, 18, 21, 22, 25, 26, 28, 29, 32, 34–38, 40–44] data collection and study appraisal (53%) [13, 17, 18, 21, 22, 27–30, 32, 34, 36–38, 42, 43] and the synthesis and findings (57%) [13, 17, 18, 21, 22, 25, 28, 29, 32, 35, 37–40, 42–44]. Only nine of the 26 non-Cochrane reviews reported the registration or existence of a review protocol [14, 19, 23, 24, 28, 30, 32, 39, 41]. The reviews used a variety of tools to assess the validity and risk of bias in trials with the Cochrane risk of bias tool being the most frequently used [6, 13–18, 20, 23–26, 29, 32, 41, 42]. Other assessment tools were, the JBL, [28, 39, 40, 45] reporting recommendations for trials of interventions for the foot in diabetes [27, 30, 46] PEDro [44, 47], the source of the risk of bias tool not reported [19, 22] QUADAS and other assessments for diagnostic tests [34, 37, 48, 49] Downs and Black [35, 50], McMaster Critical Review Form [38, 51]. Quality assessment not reported in two reviews [21, 43] preventative services veterans task force [36, 52].

**Table 2:** Risk of bias (ROBIS) assessment results

	Study eligibility criteria	Identification and selection of studies	Data collection and study appraisal	Synthesis and findings	Risk of bias in the review
Adiewere 2018 [13]	High	Unclear	Unclear	High	High
Ahmad Sharoni (2016) [29]	High	High	Unclear	High	High
Ahmed (2020) [38]	High	High	High	High	High
Alkahoon (2020) [25]	Low	Unclear	Low	Unclear	Unclear
Arad (2011) [18]	High	High	Unclear	High	High
Binning (2019) [27]	Unclear	Low	High	Low	High
Blanchette (2020) [39]	Unclear	Low	Low	High	High
Bus (2015) [32]	Low	Unclear	High	High	High
Collings (2020) [40]	Unclear	Unclear	Low	High	High
Crawford (2020) [14]	Low	Unclear	Low	Low	Unclear
Dorresteijn (2014) [15]	Low	Low	Low	Low	Low
Dy (2018) [41]	Unclear	Unclear	Low	Low	Unclear
Ena (2020) [26]	Low	Unclear	Low	Low	Unclear
Hazenberg (2019) [42]	High	High	High	High	High
He (2013) [17]	Unclear	High	Unclear	High	High
Healy (2013) [34]	Low	High	Unclear	Low	High
Heuch (2016) [28]	High	High	Unclear	High	High
Hoogeveen (2015) [16]	Low	Low	Low	Low	Low
Kaltenthaler (1998) [22]	High	High	Unclear	Unclear	High
McGloin (2021) [24]	Low	Low	Low	Low	Low
Mason (1999) [21]	High	Unclear	Unclear	Unclear	High
Matos (2018) [44]	High	High	Low	High	High
Maciejewski (2004) [36]	Unclear	High	Unclear	Low	High
Mayfield (2000) [37]	High	High	High	High	High
O’Meara (2000) [19]	Low	Low	Low	Low	Low
Norman (2020) [23]	Low	Low	Low	Low	Low
Paton (2011) [35]	Unclear	Unclear	Low	High	High
Da Silva (2020) [43]	High	High	High	High	High
Spencer (2000) [20]	Low	Low	Low	Low	Low
van Netten (2020) [30]	Low	Low	Unclear	Low	Unclear

Fifteen of the 26 non-Cochrane reviews either did not provide any information about sources of funding or declared none [17, 18, 22, 26–30, 32, 35, 37–39, 42, 43].

### Evidence of effectiveness of preventive interventions

#### Patient education

Evidence was available from four systematic reviews of patient education interventions that included four RCTs relevant to the overview [13, 15, 17, 29]. The risk of bias in the Cochrane review was judged to be low [15] while three non-Cochrane reviews were considered to be at high risk of bias [13, 17, 29].

The Cochrane review [15] published in 2014 identified two RCTs which excluded people with foot ulcers at baseline (Additional file 2: Supplementary references S1, S2). These RCTs compared intensive foot care education programmes with brief educational interventions in people at high risk of ulceration and reported contradictory results. Clinical heterogeneity precluded meta-analysis in the review as a whole which concluded there was insufficient robust evidence that patient education was effective in preventing foot ulcers.

A review comparing intensive with routine patient education published in 2013 [17] pooled the results from the same two RCTs (Additional file 2: Supplementary references S1, S2) included in the Cochrane review with results from five other trials. The meta-analysis showed a lower incidence of foot ulceration in favour of intensive education but the pooled effect estimate is unlikely to be reliable because it combined results from trials involving people with and without existing foot ulcers [53] (Additional file 2: Supplementary references S1, S2), and the authors concede some trials did not provide details of the randomisation procedure and selection bias is possible.

A subsequent review and meta-analysis [13] included six RCTs of which three met the criteria for the overview: one (Additional file 2: Supplementary reference S24) included in the Cochrane review, one (Additional file 2: Supplementary reference S3) published after completion of the Cochrane review, and interim findings from a trial (Additional file 2: Supplementary reference S4) that the Cochrane review classified as awaiting final results. One of the RCTs (Additional file 2: Supplementary reference S1) included in the previous reviews was omitted. This review was rated high for risk of bias with particular concerns about the synthesis of findings casting doubt on the reliability of the results. Meta-analysis of ulcer incidence data pooled results from trials in people with and without existing foot ulcers and failed to take account of the risk of bias in the primary studies and inconsistency in their results (I^2^ = 92%). The review’s positive conclusion, that intensive educational intervention reduced the incidence of foot ulcers compared with brief educational intervention, was based on a single meta-analysis which was interpreted as being statistically significant (*p* = 0.05). This review also pooled LEA data from dissimilar trials as reported in the earlier review by He et al. [17].

A review that intended to include only RCTs to assess the effectiveness of health education programmes to improve foot self-care and reduce foot problems in older people with diabetes expanded its scope to include non-randomised studies due to ‘the dearth of information’ identified [29]. The review method raised concerns about its ability to identify relevant studies. Ultimately it included 14 studies of various types and the only RCT (Additional file 2: Supplementary reference S2) was included in the earlier reviews we identified.

Systematic reviews that addressed the question of the effectiveness of a broad range of preventive interventions provided no additional evidence on the effectiveness of patient education from RCTs relevant to the overview. The most recent of these, an update of a previous review, undertaken to inform International Working Group on the Diabetic Foot (IWGDF) guidance on the prevention of foot ulcers in at-risk patients [30, 31] considered evidence from four RCTs (Additional file 2: Supplementary references S2, S3, S4, S14) alongside results from non-controlled studies. Conclusions were informed by a system for grading evidence-based guidelines [46] and reached by consensus. The reviews of assorted preventative interventions which included RCTs of patient education either included or pre-dated the patient education RCTs already described and identified no others [14, 18, 19, 21, 22, 25, 30, 41].

Overall these systematic reviews all found that there is inadequate evidence upon which to base recommendations about patient education to prevent foot ulceration in diabetes, [13, 15, 17, 29] except one which concluded that patient education is effective in preventing foot ulcers [13].

#### Reviews of psychosocial interventions

Three reviews assessed the evidence for psychosocial interventions to prevent foot ulcers [23, 24, 27], two of which were judged to be at low risk of bias [23, 24].

One published in the Cochrane library [24] and included a single RCT of home monitoring of foot skin temperature which included theory-based counselling for people whose foot skin temperature was raised (Additional file 2: Supplementary reference S25). A second review of psychosocial interventions included six RCTs relevant to our overview, all of which had previously been reviewed by others mostly within reviews of educational interventions [23].

A review of the effect of motivational interviewing to improve adherence behaviours for the prevention of diabetic foot ulceration was judged to be at high risk of bias [27]. The only RCT data included were the interim findings from the trial (Additional file 2: Supplementary reference S4) previously included in the review of educational interventions by Adiewere et al. [13].

These reviews all concluded there was a lack of evidence of effectiveness for psychosocial interventions or motivational interviewing and the authors of one suggested randomised controlled trials of theoretically informed interventions to assess clinical outcomes are required [23].

### Footwear and off-loading

Eight reviews [20, 28, 32, 34–36, 38, 40] aimed to evaluate footwear and/or offloading interventions and a further eight reviews of assorted interventions included footwear and offloading [14, 18, 19, 21, 22, 25, 30, 41], collectively identified nine RCTs relevant to the overview (Additional file 2: Supplementary references S5–S10, S16, S18, S19). Only two reviews were judged to be at low risk of bias [19, 20] and ten others were considered to be at high risk [18, 21, 22, 28, 32, 34–36, 38, 40].

A Cochrane review published in 2000 [20] identified one quasi-randomised trial, in which patients were allocated alternately, not randomly, showed a significant reduction in recurrent ulceration with therapeutic shoes compared with standard footwear (Additional file 2: Supplementary reference S5).

Two subsequent reviews of the effectiveness of therapeutic footwear for preventing re-ulceration [34, 36] restricted inclusion of studies to those published in English, included one additional RCT (Additional file 2: Supplementary reference S6) and other study designs. The authors concluded that the evidence to support footwear interventions to prevent re-ulceration is conflicting because non-randomised and observational studies reported positive results while the RCT showed no benefit.

The quasi-randomised trial (Additional file 2: Supplementary reference S5) was the only study with an outcome relevant to the overview that was included in a review of the effectiveness of insoles for the prevention of ulcer recurrence [35]. This review considered evidence from mixed study designs which did not support its overly positive conclusions.

A review that focussed on the effectiveness of off-loading interventions to prevent primary (first) diabetic foot ulcers was restricted to studies published in English and failed to identify any relevant RCTs with ulceration as an outcome [28].

A review [32] (updating a previous version [33]) to inform IWGDF guidance on footwear and off-loading interventions to prevent and heal diabetic foot ulcers included five additional RCTs (Additional file 2: Supplementary references S7–S11). This review considered the findings from the RCTs (including the quasi-randomised trial) alongside results from cohort studies. The authors conclude that the evidence supporting the use of specific footwear interventions to prevent recurrent plantar ulcers is quite strong and that sufficient good quality evidence supports the use of therapeutic footwear with demonstrated pressure relief to prevent plantar ulcer recurrence [32]. This finding appeared to be based on the results from a subgroup analysis within a single RCT (Additional file 2: Supplementary reference S7).

A review of the effects of footwear and insoles published in 2020 [38] identified five RCTs, only one of which had not been included in a review previously (Additional file 2: Supplementary reference S19). The RCT (*n* = 51) compared ridged with semi ridged rocker soles in people at high risk of foot ulceration and found a statistically significantly reduction in ulcers in those allocated to the ridged rocker sole. The review concluded there was limited evidence to inform the use of footwear and insoles to prevent foot ulceration. A more recent review [40] included one RCT evaluating the use of a mobile phone to alert patients of increased foot pressures which was out with the search dates of all other systematic reviews (Additional file 2: Supplementary reference S18). The proof of concept trial allocated 90 patients who were at high risk of foot ulceration to an insole system where either audio-visual alerts via a smartwatch and offloading instructions were sent to the patients’ phones when increased pressures were detected or, in the control group, no alerts were sent. The trial had a large loss to follow up (36%) and no statistically significant difference in the number of ulcerations was observed but time to event analyses found the intervention group were ulcer-free for longer. The review concludes there was difficulty in singling out the most effective weight-redistributing preventative features in shoes and insoles but concluded that this type of intervention appears to be effective.

Eight other reviews of assorted preventative interventions were identified and again either included or pre-dated RCTs of footwear and/or offloading already described and identified no others [14, 18, 19, 21, 22, 25, 30, 41]. Meta-analyses of RCT data were presented in two of the more recent reviews [14, 25]. These suggest that footwear and insoles can reduce foot ulceration but further research to examine the most effective features of footwear and insoles and their effect in people with different risk profiles is merited.

### Complex interventions

We classified three systematic reviews of the effectiveness of interventions as complex [16, 37, 39]. One review was judged to be at low risk of bias [16], two others being judged to be at high risk. There were eight reviews of assorted interventions [14, 18, 19, 21, 22, 25, 30, 41] which included integrated foot care or complex interventions, and collectively all reviews included six RCTs relevant to the overview (Additional file 2: Supplementary references S1, S13–S16, S24).

A Cochrane review published in 2015 which assessed complex interventions defined as combinations of preventive strategies identified three RCTs relevant to the overview [16]. One RCT of an education-focused intervention in low to medium-risk patients (Additional file 2: Supplementary reference S12) reported a reduction in the incidence of foot ulceration compared with usual care but may not be reliable because the cluster-randomisation design was reportedly not accounted for in the analysis. One of two RCTs that compared more intensive and comprehensive complex interventions with usual care in high-risk patients showed no difference in the incidence of foot ulceration but a significant reduction in LEA (Additional file 2: Supplementary reference S13) whereas the other trial reported the opposite (Additional file 2: Supplementary reference S14). This review judged all three RCTs at high risk of bias and the pooling of data in a meta-analysis inappropriate due to marked heterogeneity. Overall, it concluded there was insufficient evidence to support the effectiveness of complex interventions.

A review of monofilament and other threshold tests for preventing foot ulceration was judged at high risk of bias across all 4 ROBIS domains and included only one RCT evaluating the prevention of foot ulceration and amputation in people with diabetes which was also included in the Cochrane review (Additional file 2: Supplementary reference S13) [37]. The review produced overly positive conclusions about the value of screening in preventing of foot ulcers and amputations given the trial found no statistically significant difference in the incidence of foot ulcers in the two groups [37].

The same trial was excluded from a review to inform IWGDF guidance on the prevention of foot ulcers in at-risk patients because of concerns about the comparability of the intervention and control groups [30].

The review undertaken to inform IWGDF guidance included studies of integrated foot care, defined as care given by one or multiple collaborating professionals treating patients on multiple occasions with multiple interventions [30]. It excluded the trial by McCabe et al. (Additional file 2: Supplementary reference S13) but included an RCT of chiropodist care (Additional file 2: Supplementary reference S15), (which was classified in other reviews as patient education) as well as unpublished data from an additional RCT of podiatric care (Additional file 2: Supplementary reference S16) which contributed to the assessment alongside data from non-controlled studies. No conclusion could be drawn about first ulcer prevention, and the suggestion that integrated foot care may be beneficial in preventing recurrent ulceration was largely based on the results from uncontrolled studies.

A systematic review of the effect of contact with a podiatrist, working within a team, on the incidence of foot ulceration did not identify any RCTs which met its own eligibility criteria [39].

The eight reviews of assorted interventions, details of which are presented below, identified no additional trials of complex interventions [14, 18, 19, 21, 22, 25, 31, 41].

### Reviews of telehealth interventions and foot temperature monitoring

The overview identified two systematic reviews evaluating telehealth interventions to prevent foot ulceration [42, 43]. Both reviews were judged to be at high risk of bias across all 4 ROBIS domains but only one included any RCTs. The review by Hazenberg et al. [42] analysed data from 4 RCTs of home-monitoring of foot skin temperature and presented a meta-analysis showing a reduction in the number of foot ulcers when abnormal temperatures were recorded and patients’ avoided weight-bearing until their foot temperature lowered (Additional file 2: Supplementary references S21–S23, S25). These same 4 RCTs were pooled by Ena et al. [26] in a review of temperature monitoring and were also included in three systematic reviews of assorted interventions [14, 25, 30].

The two reviews conclude that further research is required, [42, 43] one also acknowledge the limitations in the studies and that a larger evidence base is required before this technology could be widely implemented in practice [42]. However, the review by Ena et al. concludes that daily measurement of skin temperature when measured using a handheld infrared thermometer reduces the appearance of new foot ulcers and notes the risk of bias in the same 4 RCTs is low (Additional file 2: Supplementary references S21–S23, S25) [26]. The three reviews of assorted interventions all concluded that the available data suggest this intervention may prevent foot ulcers developing [14, 25, 30] but two noted the need for further evaluation and the possibility that the intervention might not be feasible in real world settings [14, 25].

### Reviews of physical activity

We found one systematic review of physical activity which we judged to be at high risk of bias in its evaluation of the effect of exercise of the prevention of foot ulceration [44]. It included one RCT in which foot ulceration was an outcome (Additional file 2: Supplementary reference S24). The reviewers’ conclusion that exercise can delay the development of foot ulcers is not supported by the trial results (Additional file 2: Supplementary reference S24). The RCT was also included in three separate systematic reviews of assorted interventions [14, 30, 41].

### Reviews of assorted preventative interventions

Eight systematic reviews included a variety of interventions to prevent foot ulcers [14, 18, 19, 21, 22, 25, 30, 41] only one was judged to be at low risk of bias [19]. Four were judged to have an unclear risk of bias because of approaches they took to the selection of studies or the analysis [14, 25, 30, 41] and three were judged at high risk of bias [18, 21, 22].

Collectively they assessed the evidence from 26 RCTs, 2 of which were not included in intervention-specific reviews presented above. Two reviews included a trial of elastic compression stockings as a preventive intervention [14, 19]. The incidence of foot ulcers in people randomised to elastic compression stockings compared with those who did not receive hosiery was not found to be statistically significantly different. The trial population was at high risk of foot ulceration (Additional file 2: Supplementary reference S26).

Three reviews [14, 31, 41] included one RCT of patient instruction to apply antifungal nail lacquer as a way to increase the frequency of foot self-inspection but found no difference in the incidence of first or recurrent ulcers when compared with standard care (Additional file 2: Supplementary reference S17).

## Discussion

Systematic reviews are widely regarded as the cornerstone of evidence-based healthcare. Harnessing that evidence has become increasingly challenging as the prevalence of systematic reviews in the biomedical literature continues to increase with one recent estimate suggesting a publication rate of more than 8000 per year [9]. It is therefore unsurprising that we identified 30 systematic reviews of interventions to prevent diabetic foot ulceration that met the criteria for inclusion in our overview, with one-third having been published in the last 5 years. Yet, this surfeit of systematic reviews does not provide a wholly reliable source of evidence for decision-making.

The ability of an overview to provide useful decision-support is reliant on the quality of the conduct and reporting of the systematic reviews available. As stated, our original purpose was to conduct an overview of reviews to obtain numerical summaries of the effects of preventative interventions for foot ulcers in diabetes to populate an economic model, but two-thirds of the reviews we included had methodological shortcomings associated with a high risk of bias and reliable meta-analyses of trial data were first published in 2020 [14, 25].

Those reviews without protocols made it difficult to ascertain whether the reviews’ methods were pre-defined, adhered to or decided or modified during the review process. The absence of pre specified primary study inclusion criteria in a third of the reviews also made it hard to judge whether reviewers’ decisions about including studies during the conduct of the reviews could have introduced bias. The evident inadequate development of search strategies may suggest a lack of familiarity with the principles of searching electronic databases and working with an information specialist who possesses the skills to construct and implement robust search strategies. Searches were frequently compromised by involving few sources, limited search terms and unjustified restrictions. Only around half of the reviews searched without language restrictions and few searched sources of unpublished data. More than half of the reviews included various study designs as well as RCTs but few considered the influence that study design could have on the results.

The conduct of evidence synthesis was another common cause for concern about bias in most of the reviews we identified. Quantitative synthesis of RCT data was performed in only five of the reviews but we found problems with meta-analyses that included data from patients who did not meet predefined eligibility criteria, errors in the interpretation of meta-analytical statistics and failure to explore reasons for heterogeneity. Narrative approaches largely entailed study-by-study narrative summaries which may indicate a lack of awareness or expertise in methods for the conduct of narrative synthesis in systematic reviews. Whatever the approach used, interpretation of the findings often ignored or glossed over the potential for bias in the included studies and other important between-study differences. The upshot of this is seen in overly positive conclusions that are not supported by the evidence reviewed.

Guidance for conducting overviews is accumulating but challenges remain [54] and some limitations to our overview warrant consideration. We could have missed some relevant systematic reviews by not searching a wider range of sources but, finding more reviews is unlikely to have altered our concern about the reliability of the evidence base as a whole. We may also have failed to find reviews including RCTs of other relevant interventions. We used ROBIS [12] to appraise the quality of the included systematic reviews but found that using this validated tool often relied on subjective judgment, especially in the absence of review protocols, resulting in lengthy deliberations to resolve disagreements. Research published by others has shown inadequate inter-rater reliability among professional reviewers using ROBIS [55] and we concur that the tool and guidance need revision to improve its reliability and utility. We suggest that reviewers who intend to use ROBIS to assess the risk of bias in systematic reviews clarify and agree the reasons for allocating specific ratings during the development of the protocol and again periodically during the conduct of the overview.

In any overview of multiple systematic reviews evaluating alternative intervention options some overlap in the included primary studies is to be expected and has to be assessed to avoid introducing bias [56, 57]. This overview revealed how substantial the overlapping nature of the evidence from systematic reviews of RCTs addressing diabetic foot ulcer prevention is and crucially, the same (largely poor-quality) trials being reviewed over and over again without our understanding about what works to improve patient outcomes becoming any clearer [14].

The predominance of low-quality trials that are subsequently included in systematic reviews without due consideration is a concern for journal editors as it undermines confidence in systematic reviews to reliably inform clinical practice [58]. From the overview it appears some editors do not share those concerns and may not even be aware of the methodological flaws in the systematic reviews their journals have published. This is at odds with the endorsement of the Preferred Reporting Items for Systematic Reviews and Meta-analyses (PRISMA) reporting guidelines [59] by most of those journals in their instructions to authors and is also hard to reconcile with a robust peer review process. These systematic reviews were published over two decades, but we saw little improvement in the quality of conduct and reporting over time. This mirrors the pattern observed more widely in the biomedical literature by researchers who have recommended certified training for journal editors in how to implement PRISMA and facilitate its use by peer reviewers as one way to improve the value of systematic reviews [9]. The same challenges might also exist for the recent reporting guidelines for literature searches in systematic reviews, PRISMA-S, despite the clear intention to improve the reproducibility of searches in reviews [60].

Practitioners involved in developing international guidelines on the prevention of diabetic foot ulcers recognise the need to improve the quality of the intervention studies that are conducted and submitted for publication [46]. They have drawn attention to the omission of core details from many trial reports that hinders appraisal of study quality and clinical relevance in systematic reviews. This has implications for relying on overviews to understand the evidence base if it is not possible to tell from systematic review reports whether missing details were absent from the included trial reports or overlooked by the reviewers. The proposed reporting standards checklist for studies on the management and prevention of foot ulcers in diabetes should inform the conduct of systematic reviews as well primary studies alongside PRISMA and CONSORT (Consolidated Standards of Reporting Trials) [59, 61] to improve the quality of published research in this area. Other researchers engaged in synthesizing evidence of health technologies for clinical conditions other than the foot in diabetes may also find condition-specific reporting standards helpful when undertaking an assessment of relevant literature.

Using evidence from unreliable systematic reviews to inform clinical practice has obvious negative consequences including invalid clinical guidelines recommendations which could result in the provision of suboptimal care that will not lead to improved outcomes for patients. There is already evidence that the number of overviews of systematic reviews of healthcare interventions is rising and their quality is variable [62]. Given the abundance of systematic reviews summarising poor-quality trials of interventions to prevent diabetic foot ulcers, it may only be a matter of time before uncritical overviews also start to proliferate. Those who conduct, fund, peer review and publish research in this area have a joint responsibility to ensure that the evidence base does not serve the interests of researchers and publishers rather than improving outcomes for people living with diabetes.

### Supplementary Information

**Additional file 1.**

**Additional file 2.**

## Data Availability

All data generated or analysed during the current study are included in this published article and its supplementary information files.

## Appendix

### Medline strategy

1. exp foot orthoses/
2. exp shoes/
3. exp health education/
4. exp primary health care/
5. exp emollients/
6. insole*.mp.
7. footwear.mp.
8. educat*.mp.
9. specialist car*.mp.
10. multi disciplinary team*.mp.
11. multidisciplinary team*.mp.
12. routine podiatry car*.mp.
13. exp general practice/
14. exp community health services/
15. off load*.mp.
16. offload*.mp.
17. emollient*.mp.
18. shoe*.mp.
19. or/1–18
20. exp foot/
21. exp foot diseases/
22. exp diabetic foot/
23. exp diabetic neuropathies/
24. exp diabetes mellitus/
25. exp diabetic angiopathies/
26. exp diabetes complications/
27. exp podiatry/
28. exp foot ulcer/
29. exp skin ulcer/
30. exp ischemia/
31. exp bacterial infections/
32. (diabet* adj3 ulcer*).mp.
33. (diabet* adj3 (foot or feet)).mp.
34. (diabet* adj3 wound*).mp.
35. (diabet* adj3 amputat*).mp.
36. or/20–35
37. systematic* review*.mp.
38. meta-analysis as topic/
39. (meta-analytic* or meta-analysis or metanalysis or metaanalysis or meta analysis or meta synthesis or meta-synthesis or metasynthesis or meta-regression or metaregression or meta regression).mp.
40. (synthes* adj3 literature).mp.
41. (synthes* adj3 evidence).mp.
42. (integrative review or data synthesis).mp.
43. (research synthesis or narrative synthesis).mp.
44. (systematic study or systematic studies).mp.
45. (systematic comparison* or systematic overview*).mp.
46. ((evidence based or comprehensive or critical or quantitative or structured) adj review).mp.
47. (realist adj (review or synthesis)).mp.
48. or/37–47
49. review.pt.
50. (medline or pubmed or embase or cinahl or psyc?lit or psyc?info).mp.
51. ((literature or database* or bibliographic or electronic or computeri?ed. or internet) adj3 search*).mp.
52. (electronic adj3 database*).mp.
53. included studies.mp.
54. (inclusion adj3 studies).mp.
55. ((inclusion or selection or predefined or predetermined) adj criteria).mp.
56. (assess* adj3 (quality or validity)).mp.
57. (select* adj3 (study or studies)).mp.
58. (data adj3 extract*).mp.
59. extracted data.mp.
60. (data adj3 abstraction).mp.
61. published intervention*.mp.
62. ((study or studies) adj2 evaluat*).mp.
63. (intervention* adj2 evaluat*).mp.
64. (confidence interval* or heterogeneity or pooled or pooling or odds ratio*).mp.
65. (Jadad or coding).mp.
66. or/50–65
67. 49 and 66
68. review.ti.
69. 66 and 68
70. (review* adj4 (papers or trials or studies or evidence or intervention* or evaluation*)).mp.
71. 48 or 67 or 69 or 70
72. letter.pt.
73. editorial.pt.
74. comment.pt.
75. 72 or 73 or 74
76. 71 not 75
77. 19 and 36 and 76

### Embase search strategy

1. exp foot orthosis/
2. shoe/
3. exp health education/
4. exp primary health care/
5. emollient agent/
6. insole*.mp.
7. footwear*.mp.
8. educat*.mp.
9. specialist car*.mp.
10. multi disciplinary team*.mp.
11. multidisciplinary team*.mp.
12. routine podiatry car*.mp.
13. general practice/
14. exp community care/
15. off load*.mp.
16. offload*.mp.
17. emollient*.mp.
18. shoe*.mp.
19. 1 or 2 or 3 or 4 or 5 or 6 or 7 or 8 or 9 or 10 or 11 or 12 or 13 or 14 or 15 or 16 or 17 or 18
20. exp foot/
21. exp foot disease/
22. diabetic foot/
23. diabetic neuropathy/
24. exp diabetes mellitus/
25. exp diabetic angiopathy/
26. (diabet* adj3 complicat*).mp.
27. podiatry/
28. foot ulcer/
29. exp skin ulcer/
30. exp ischemia/
31. exp bacterial infection/
32. (diabet* adj3 ulcer*).mp.
33. (diabet* adj3 (foot or feet)).mp.
34. (diabet* adj 3 wound*).mp.
35. (diabet* adj3 amputat*).mp.
36. 20 or 21 or 22 or 23 or 24 or 25 or 26 or 27 or 28 or 29 or 30 or 31 or 32 or 33 or 34 or 35
37. systematic* review*.mp.
38. meta analysis/
39. (meta-analytic* or meta-analysis or metanalysis or metaanalysis or meta analysis or meta synthesis or meta-synthesis or metasynthesis or meta-regression or metaregression or meta regression).mp.
40. (synthes* adj3 literature).mp.
41. (synthes* adj3 evidence).mp.
42. (integrative review or data synthesis).mp.
43. (research synthesis or narrative synthesis).mp.
44. (systematic study or systematic studies).mp.
45. (systematic comparison* or systematic overview*).mp.
46. ((evidence based or comprehensive or critical or quantitative or structured) adj review).mp.
47. (realist adj (review or synthesis)).mp.
48. or/37–47
49. review.pt.
50. (medline or pubmed or embase or cinahl or psyc?lit or psyc?info).mp.
51. ((literature or database* or bibliographic or electronic or computer?ed. or internet) adj3 search*).mp.
52. (electronic adj3 database*).mp.
53. included studies.mp.
54. (inclusion adj3 studies).mp.
55. ((inclusion or selection or predefined or predetermined) adj criteria).mp.
56. (assess* adj3 (quality or validity)).mp.
57. (select* adj3 (study or studies)).mp.
58. (data adj3 extract*).mp.
59. extracted data.mp.
60. (data adj3 abstraction).mp.
61. published intervention*.mp.
62. ((study or studies) adj2 evaluat*).mp.
63. (intervention* adj2 evaluat*).mp.
64. (confidence interval* or heterogeneity or pooled or pooling or odds ratio*).mp.
65. (jadad or coding).mp.
66. or/50–65
67. 49 and 66
68. review.ti.
69. 66 and 68
70. (review* adj4 (papers or trials or studies or evidence or intervention* or evaluation*)).mp.
71. 48 or 67 or 69 or 70
72. letter.pt.
73. editorial.pt.
74. comment.pt.
75. 72 or 73 or 74
76. 71 not 75
77. exp. animals/not humans/
78. 78.76 not 77
79. 19 and 36 and 76
