# Supplementary material for: Reliability of the evidence to guide decision-making in foot ulcer prevention in diabetes: an overview of systematic reviews
Source: BMC Med Res Methodol. 2022 Oct 20;22:274. doi: 10.1186/s12874-022-01738-y (PMC9583498; doi:10.1186/s12874-022-01738-y)
Supplement: Supplementary file 2 — Additional file 2. [file 12874_2022_1738_MOESM2_ESM.docx]

Supplementary references – Randomised controlled trials in the systematic reviews

S1. Cisneros LL. Evaluation of a neuropathic ulcers prevention program for patients with diabetes [Avaliação de um programa para prevenção de úlceras neuropáticas em portadores de diabetes]. Rev Bras Fisioter. 2010;14(1):31–7.

S2. Lincoln NB, Radford KA, Game FL, Jeffcoate WJ. Education for secondary prevention of foot ulcers in people with diabetes: a randomised controlled trial. Diabetologia. 2008;51(11):1954–1961.

S3. Monami M, Zannoni S, Gaias M, Nreu B, Marchionni N, Mannucci E. Effects of a short educational program for the prevention of foot ulcers in high-risk patients: a randomized controlled trial. Int J Endocrinol. 2015;615680:1e5.

S4. Gershater M, Pilhammar E, Apelqvist J, Alm‐Roijer C. Patient education for the prevention of diabetic foot ulcers. Interim analysis of a randomised controlled trial due to morbidity and mortality of participants. Eur Diabetes Nurs. 2011;8(3):102‐107b.

S5. Uccioli L, Faglia E, Monticone G, Favales F, Durola L, Aldeghi A, Quarantiello A, Calia P, Menzinger G. Manufactured shoes in the prevention of diabetic foot ulcers. Diabetes Care. 1995;18(10):1376–8.

S6. Reiber GE, Smith DG, Wallace C, Sullivan K, Hayes S, Vath C, Maciejewski ML, Yu O, Heagerty PJ, LeMaster J. Effect of therapeutic footwear on foot reulceration in patients with diabetes: a randomized controlled trial. JAMA. 2002;287(19):2552–2558.

S7. Bus SA, Waaijman R, Arts M. Effect of custom-made footwear on foot ulcer recurrence in diabetes.: a multicentre randomised controlled trial. Diabetes Care 2013;36(12):4109-4116.

S8. Ulbrecht JS, Hurley T, Mauger DT, Cavanagh PR. Prevention of recurrent foot ulcers with plantar pressure-based in-shoe orthoses: the CareFUL prevention multicenter randomized controlled trial. Diabetes Care. 2014;37(7):1982–9.

S9. Lavery L, LaFontaine J, Higgins K, Lanctot D, Constantinides G. Shear-reducing insoles to prevent foot ulceration in high-risk diabetic patients. Adv Ski Wound Care. 2012;25(11):519–524.

S10. Rizzo L, Tedeschi A, Fallani E, Coppelli A, Vallini V, Iacopi E, Piaggesi A. Custom-made orthesis and shoes in a structured follow-up program reduces the incidence of neuropathic ulcers in high-risk diabetic foot patients. Int J Low Extrem Wound. 2012;11(1):59–64.

S11. Scire V, Leporati E, Teobaldi I, Nobili LA, Rizzo L, Piaggesi A. Effectiveness and safety of using Podikon digital silicone padding in the primary prevention of neuropathic lesions in the forefoot of diabetic patients. J Am Pod Med Assoc. 2009;99(1):28–34.

S12. Litzelman DK, Slemenda CW, Langefeld CD, Hays LM, Welch MA, Bild DE, Ford ES, Vinicor F. Reduction of lower extremity clinical abnormalities in patients with non-insulin-dependent diabetes mellitus. Ann Intern Med. 1993;119(1):36–41.

S13. McCabe CJ, Stevenson RC, Dolan AM. Evaluation of a diabetic foot screening and protection programme. Diabet Med. 1998;15(1):80–4.

S14. Liang R, Dai X, Zuojie L, Zhou A, Meijuan C. Two-year foot care program for minority patients with type 2 diabetes mellitus of Zhuang Tribe in Guangxi, China. Can J Diabetes. 2012;36(1):15–18.

S15. Plank J, Haas W, Rakovac I, Görzer E, Sommer R, Siebenhofer A, Pieber TR. Evaluation of the impact of chiropodist care in the secondary prevention of foot ulcerations in diabetic subjects. Diabetes Care. 2003;26(6):1691–5.

S16 Van Putten M. The effectiveness of a preventive foot care program versus treatment as usual to reduce the number of ulcers in diabetic patients with polyneuropathy: a randomized controlled trial [Internet]. ISRCTN Registry; Available from: <https://doi.org/10.1186/ISRCTN50646165>

S17. Armstrong DG, Holtz K, Wu S. Can the use of a topical antifungal nail lacquer reduce risk for diabetic foot ulceration? Results from a randomised controlled pilot study. Int Wound J. 2005;2(2):166–70.

S18. Abbott CA, Chatwin KE, Foden P, Hasan AN, Sange C, Rajbhandari SM, Reddy PN, Vileikyte L, Bowling FL, Boulton AJM, Reeves ND. Innovatived intellegent insole system reduces diabetic foot ulcer recurrence at plantar sites: a prospective, randomised, proof of concept study. Lancet Digital Health 2019; 1: e308–18

S19. Lopez-Moral M, Lazaro-Martinez JL, Garcia-Morales E, Garcia-Alvarez Y, Alvaro-Alfonzo FJ, Molines-Barroso RJ. Clinical Efficacy of therapeutic footwear with a rigid rocker sole in the prevention of recurrence in pateints with diabetes mellitus and diabetic polineuropathy: A randomised clinical trial. PLOS ONE; 14(7):e0219537. https:// doi.org/10.1371/journal.pone.0219537.

S20. Liu J, Chen T, Wang S, Liu H. The effects of transitional care on the prevention of foot ulcers in patient at high risk for diabetic foot. International Journal of Diabetes in Developing Countries (October–December 2019) 39(4):659–666. DOI.ORG.1007/s13410-019-00736-z.

S21. Armstrong DG, Holtz-Neiderer K, Wendel C, Mohler MJ, Kimbriel HR, Lavery LA. Skin temperature monitoring reduces the risk for diabetic foot ulceration in high-risk patients. Am J Med. 2007;120(12):1042–6.

S22. Lavery LA, Higgins KR, Lanctot DR, Constantinides GP, Zamorano RG, Athanasiou KA, Armstrong DG, Agrawal CM. Preventing diabetic foot ulcer recurrence in high-risk patients: use of temperature monitoring as a self-assessment tool. Diabetes Care. 2007;30(1):14–20.

S23. Lavery L, Higgins K, Lanctot D, Constantinides G, Zamorano R, Armstrong D, Athanasiou KA, Agrawal CM. Home monitoring of foot skin temperatures to prevent ulceration. Diabetes Care. 2004;27(11):2642–2647.

S24 LeMasterJW, Mueller MJ, Reiber GE, Mehr DR, Madsen RW, Conn VS,. Effect of weight-bearing activity on foot ulcer incidence in people with diabetic peripheral neuropathy: feet first randomized controlled trial. Physical Therapy 2008;88(11):1385-1398.

S25 Skafjeld A, Iveren MM, Holme I, Ribu L, Kilhovd BK. A pilot study testing temperature monitoring to reduce recurrent foot ulcers in patients with diabetes – a randomized controlled trial. BMC Endocrine Disorders15:55.

S26 Belcaro G, Laurora G, Cesarone MR, Pomante P,. Elastic stockings in diabetic macroangiopathy. Long term clinical and microcirculatory evaluation. Vasa 21(2):193-197.
